# Arterial blood flow detection and clinical outcomes of patients with flat spots and diverticular hemorrhage

**DOI:** 10.1016/j.igie.2025.10.015

**Published:** 2025-10-25

**Authors:** Peerapol Wangrattanapranee, Dennis M. Jensen, Mary Ellen Jensen, Gail Thibodeau

**Affiliations:** 1 VA GI Hemostasis Research Unit, VA Greater Los Angeles Healthcare System, Los Angeles, California, USA; 2 Division of Gastroenterology and Hepatology, Department of Internal Medicine, University of California, Riverside, California, USA; 3 Gastroenterology Division, Department of Medicine, VA Greater Los Angeles Healthcare System, Los Angeles, California, USA; 4 Vatche and Tamar Manoukian Division of Digestive Diseases, Department of Medicine, Ronald Reagan UCLA Medical Center, Los Angeles, California, USA; 5 David Geffen School of Medicine at University of California, Los Angeles, California, USA

## Abstract

**Background and Aims:**

Colonoscopic hemostasis is recommended for diverticular (TIC) hemorrhage (TICH) with major stigmata of recent hemorrhage (SRH). Whether flat spots should be included as a major SRH is unknown. For TIC flat spots, our aims were (1) to report rates of blood flow detected underneath by Doppler endoscopic probe (DEP), (2) to compare rebleeding rates of medical versus endoscopic treatment, and (3) to compare rates of arterial blood flow detection and rebleeding with nonbleeding visible vessels (NBVVs).

**Methods:**

This is a retrospective study of prospectively collected data of patients at West Los Angeles Veterans Affairs and Ronald Reagan University of California, Los Angeles Medical Centers (Los Angeles, Calif, USA) from 1993 to 2023 for definitive TICH from either flat spots or NBVVs. Arterial blood flow underneath SRH was monitored by DEP. Descriptive and comparative analyses were used.

**Results:**

Of 24 patients with TIC flat spots, treatment in 83.3% was hemostatic clips versus 16.7% medical. Seventeen of 19 patients with DEP monitoring had positive arterial blood flow, and all had hemostatic clipping. Within 30 days, 25% of the medical group rebled, whereas 10% of the endoscopic group rebled. After 30 days, 35% of the endoscopic group rebled versus none of the medical group. Rates of positive DEP were the same for flat spots and NBVVs. Flat spot patients had a significantly lower rate of colonoscopic hemostasis and a higher rebleeding rate.

**Conclusions:**

TIC flat spots should be considered as a major SRH and treated because of high rates of arterial blood flow underneath and rebleeding. Improvements in TICH spot hemostasis are warranted.

## Introduction

Diverticular (TIC) hemorrhage (TICH) is the most common cause of severe acute lower gastrointestinal (GI) hemorrhage (LGIH) in adults.^1^ The highest prevalence of TICH is 63% reported in Japan,^2^ but it was diagnosed in only 32.5% in the United States and 26.4% in the United Kingdom of all acute LGIH.^3^^,^^4^ Several factors could contribute to these differences, including how LGIH is defined and diagnosed, patient populations, gastroenterologist's expertise, what tests were used for diagnosis, how coding of TICH was done, timing of colonoscopy, and quality of bowel preparation. Nevertheless, recognizing TIC stigmata of recent hemorrhage (SRH), their risks for TIC rebleed, and the best management strategy are crucial in improving clinical outcomes.

The Center for Ulcer Research and Education (CURE) Hemostasis Research Group previously classified TICH into 3 groups: definitive TICH, presumptive TICH, and incidental TIC.^5^ Definitive TICH was defined when an SRH was found in a single TIC. The classification of SRH in TICH was similar to peptic ulcer bleeding (PUB). Major SRH included active bleeding, nonbleeding visible vessels (NBVVs), or adherent clot. Flat spots, flat hyperpigmented lesions without active bleeding, visible vessel, or adherent clot were not included as a major SRH.^5^ Presumptive TICH was defined when no SRH was found and no other sources of GI bleeding were identified. Incidental TIC was defined when other sources of GI tract were identified, but TICs were found on colonoscopy.^6^

Prospective studies reported the importance of detecting and treating TIC SRH because colonoscopic hemostasis significantly reduced the risk of TIC rebleeding and improved patient outcomes.^5^, ^6^, ^7^, ^8^, ^9^ Our group previously recommended treating major SRH of TICH including active bleeding, NBVVs, or adherent clot.^5^^,^^10^ Another previous study showed that 67% of flat spots were positive for arterial blood flow by Doppler endoscopic probe (DEP), and the rebleeding rate was 60% with medical treatment alone, similar to other major TIC SRH.^11^^,^^12^

Our study aims were (1) to report rates of arterial blood flow detection by DEP under TIC flat spots, (2) to compare rebleeding rates of patients with flat spots treated medically versus endoscopically, and (3) to compare rates of blood flow detection by DEP and rebleeding for patients with TIC flat spots or NBVVs.

## Methods

### Study design, setting, and patient population

This was a retrospective cohort study of prospectively collected data at the West Los Angeles Veterans Affairs and Ronald Reagan University of California, Los Angeles Medical Centers (Los Angeles, Calif, USA) from 1993 to 2023. All patients were hospitalized with severe hematochezia and had definitive TICH with either flat spots or NBVVs during urgent colonoscopy. Severe hematochezia was defined as a decrease of hemoglobin (Hb) at least 2 g/dL and/or at least 1 unit of packed red blood cell transfusion. All patients were included in Institutional Review Board—approved studies of severe hematochezia.

### Management of definitive TICH

On hospitalization, all patients were resuscitated and transfused if Hb was below 7 or 8 g/dL if they had severe cardiovascular disease. Aspirin was withheld in patients taking it for primary cardiovascular prevention. For patients on antiplatelet therapy and/or anticoagulants due to a history of cerebro- or cardiovascular diseases, the decision to discontinue these was made after consulting with specialists. Prothrombin complex concentrate or fresh frozen plasma was reserved for those with severe coagulopathy and life-threatening bleeding.^13^

After 6 L to 8 L of a polyethylene glycol solution, urgent colonoscopy was performed within 24 hours by a team of experienced gastroenterologists. DEP became available at our hospitals in 2007 for detecting arterial blood flow underneath SRH (Fig. 1). The DEP monitoring device (Vascular Technology Inc, Nashua, NH, USA) consists of a control unit and 2-mm-diameter disposable catheter inserted through the suction channel of the colonoscope. Arterial blood flow was detected by a swish-swish sound from the control unit using gated settings of superficial (<1.5 mm) or medium depth (1.5-4 mm). DEP was performed after colonoscopic hemostasis to confirm successful arterial obliteration (Fig. 2).

**Figure 1:**
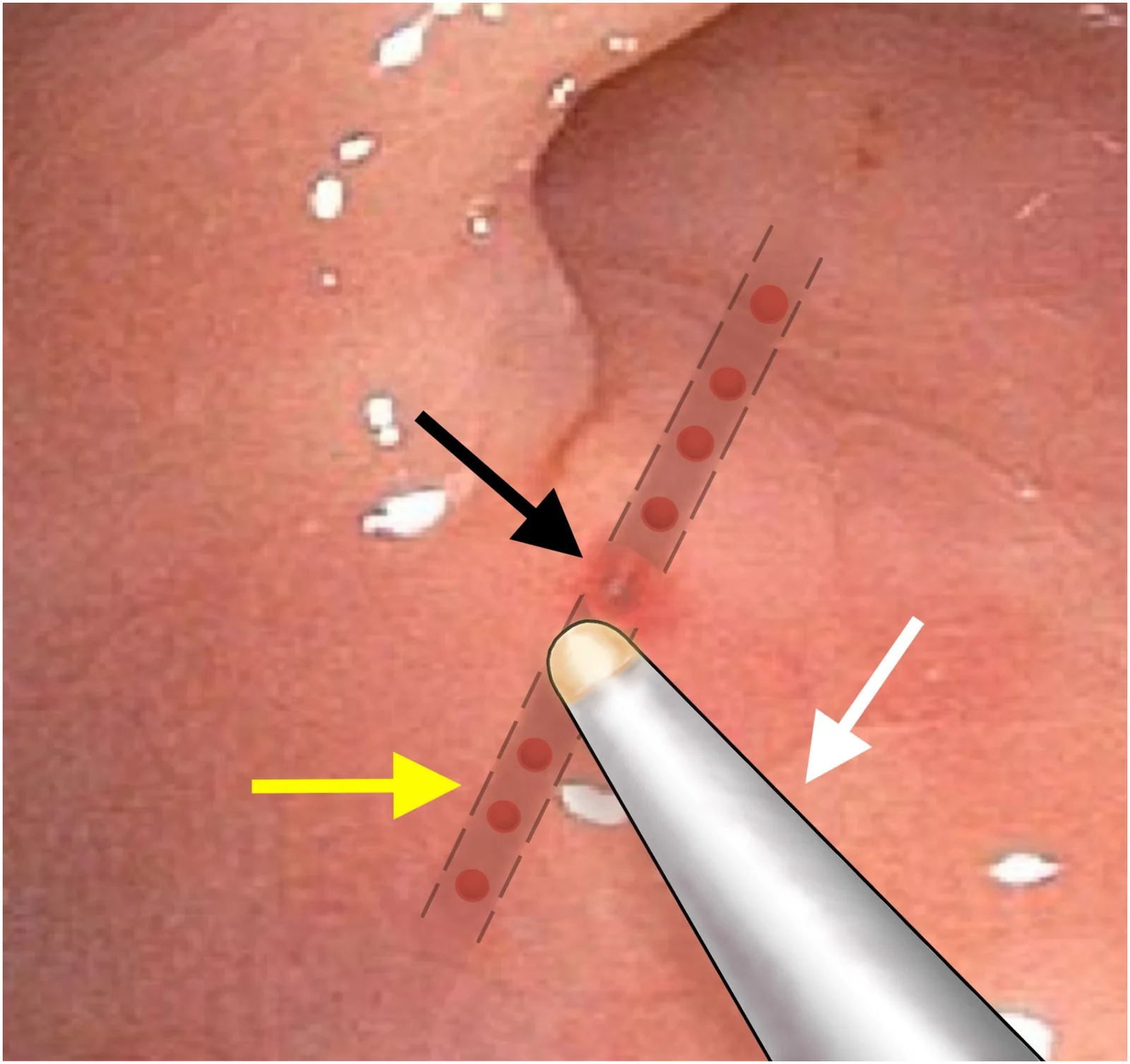
Diagram of Doppler endoscopic probe interrogation for arterial blood flow on the flat spot prior to hemostasis (*yellow arrow* = artery underneath flat spot, *black arrow* = flat spot, *white arrow* = Doppler endoscopic probe).

**Figure 2:**
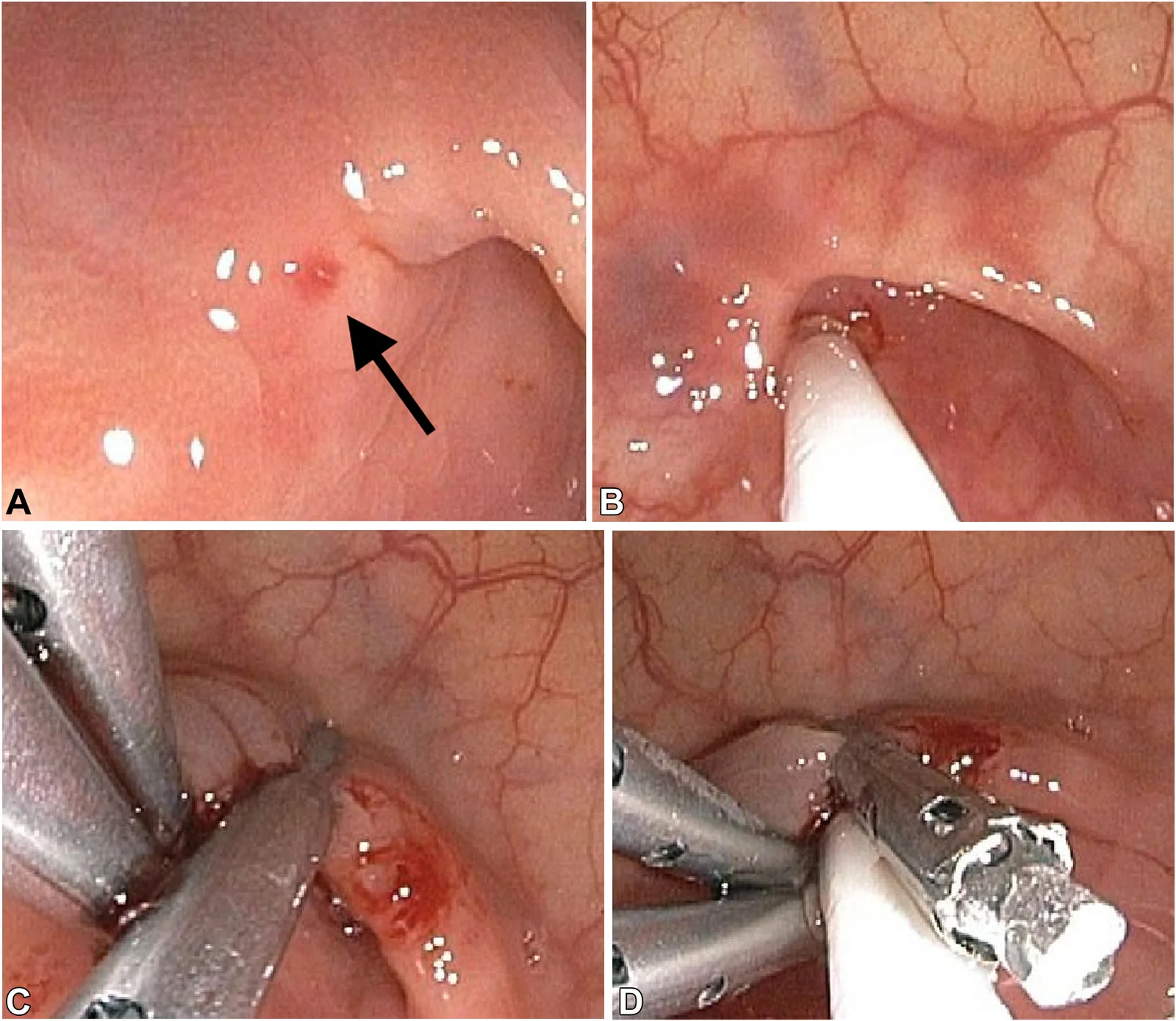
Doppler endoscopic probe–guided treatment of diverticular flat spot. **A,** Flat spot at the neck of a diverticulum (*arrow*). **B,** Doppler endoscopic probe interrogation of the flat spot. **C,** Three hemostatic clips deployed—1 on each side of the flat spot and 1 on the flat spot. **D,** Repeat Doppler endoscopic probe interrogation after hemostatic clips to document obliteration of arterial blood flow.

In the medical group, all patients received treatment as described. In the endoscopic group, all patients received the same medical treatment with additional colonoscopic hemostasis. Hemostatic clipping was performed for all flat spot cases including those at TIC neck and base (Figure 1, Figure 2, Figure 3). Figure 2 presents a case example of a flat spot at the TIC neck with DEP monitoring before and after hemostatic clipping. Then, India ink solution was used for tattooing in 4 quadrants around the TIC to facilitate TIC localization during recurrent LGIH.^10^^,^^12^

**Figure 3:**
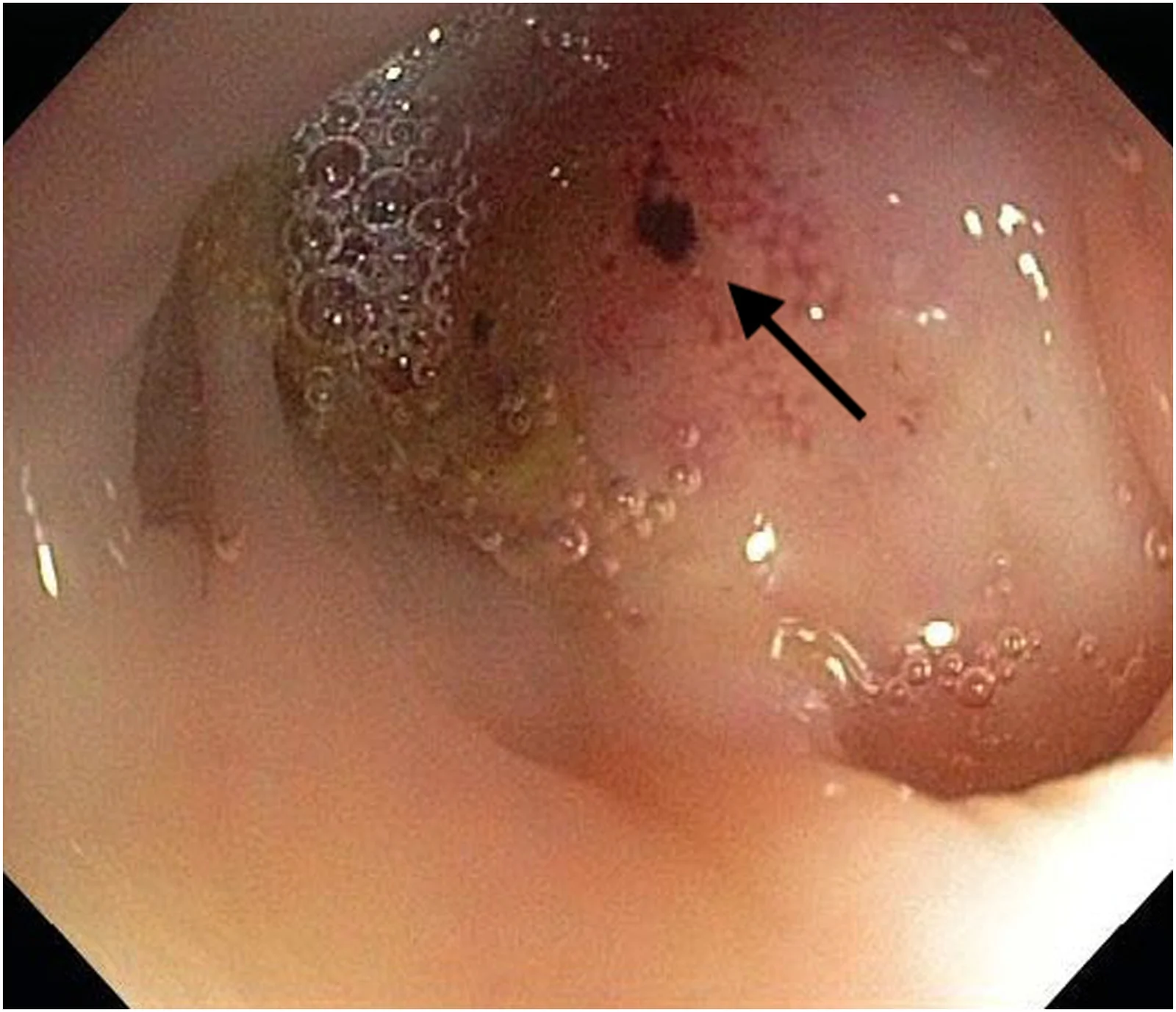
Flat spot at the base of a diverticulum (*arrow*).

For some NBVV cases at the neck of the TIC (Fig. 4), multipolar electrocoagulation (MPEC) with low power (12-14 W), short pulses (1-2 seconds), and moderate laterally applied tamponade pressure was performed.^10^^,^^11^ Other NBVV cases were treated with hemostatic clipping, MPEC, or a combination of both.^10^^,^^11^

**Figure 4:**
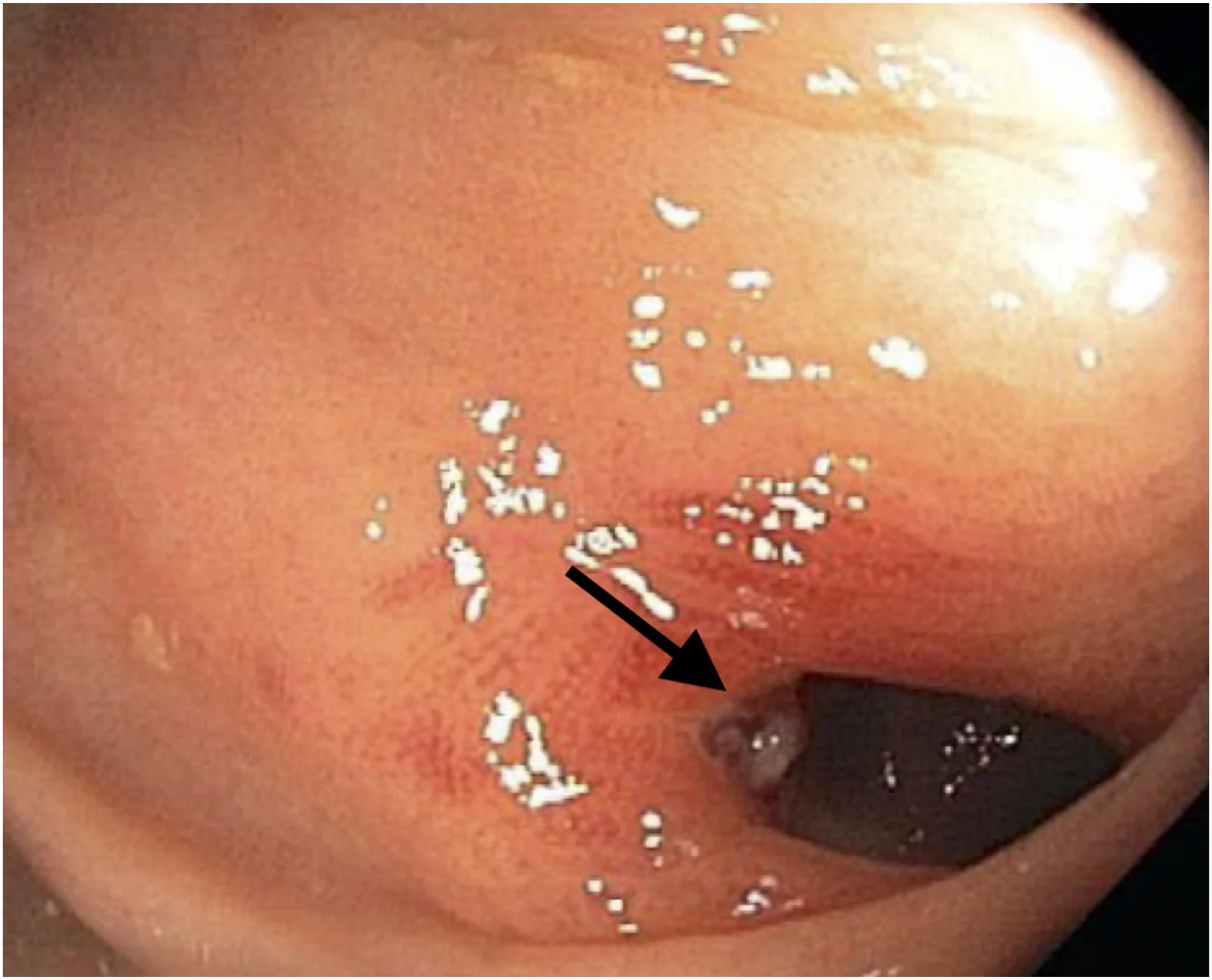
Nonbleeding visible vessel at the neck of a diverticulum (*arrow*).

On discharge, fiber supplement and medications to control constipation were prescribed for all patients. Antithrombotic drugs were restarted based on recommendations by specialists within 5 days of the index TIC bleed.

### Data collection and follow-up after hospitalization

Data collected were demographics, bleeding medications, CURE prognosis score (1 point for each of the following: age ≥60 years, systolic blood pressure <100 or severe hemodynamic instability, ≥5 units of packed RBC, rebleeding in the hospital, presence of other comorbidities, severe comorbidities making prognosis poor), Glasgow-Blatchford score, Charlson Comorbidity Index, lowest Hb, packed RBC transfusion, types and locations of SRH, DEP use, and endoscopic treatment. The left-sided colon was defined when TIC SRH was located from the splenic flexure to the rectum. The right-sided colon was defined from the cecum to the splenic flexure. After patients’ discharge from the hospital, our GI research team prospectively collected outcome data every month for the first 3 months, then every 3 months for the first year, and then annually. Rebleeding was defined as the same as the index TICH.

### Outcomes

Primary outcomes were rates of DEP positivity of arterial blood flow underneath flat spots and early (≤30 days) rebleeding from the same TIC. The secondary outcome was the rate of late (>30 days) rebleeding from the same TIC. Other analyses were performed to compare rebleeding rates of patients with spots in the TIC base versus neck, as well as to compare rates of DEP positivity from spots versus NBVV and TIC rebleeding rates.

### Statistical analysis

Categorical variables were reported as numbers and percentages and compared using χ^2^ or Fisher exact tests. Continuous variables with normal distribution were reported as mean and standard deviation and compared using the *t* test. Continuous variables with nonnormal distribution were reported as median and interquartile range (IQR) and compared using the Mann-Whitney U test. *P* values less than .05 were considered statistically significant. Statistical analysis was performed by IBM SPSS Statistics for Macintosh, version 29.0.2.0 (IBM Corp, Armonk, NY, USA).

## Results

### TIC patients with flat spots

#### Patients' clinical characteristics

Of 24 patients with TIC flat spots, 75% were male and 25% female. The mean age was 73 years. The most common races were African American and Caucasian (n = 8 for each), followed by Asian (n = 4) and Hispanic (n = 4). Aspirin use was high (50%), followed by nonsteroidal anti-inflammatory drugs (NSAIDs) (16.7%), antiplatelet drugs (12.5%), and anticoagulants (8.3%). Fifty-four percent had prior TICH, either presumptive or definitive.

All patients presented with severe hematochezia, but only 1 patient had shock, and 5 patients had hypotension. Two-thirds (66.7%) received packed RBC transfusions, and the mean Hb was 8.3 mg/dL. Furthermore, 37.5% had a history of chronic anemia and 8.3% had coagulopathies. The median (IQR) CURE prognosis score was 3 (1), the Charlson Comorbidity Index was 4 (5), and the Glasgow-Blatchford score was 8 (4).

#### Antithrombotic management

One patient on Apixaban for pulmonary embolism had medication withheld, but it was not resumed because of massive GI bleed. This patient was started on warfarin 1 month later because of new deep vein thrombosis.

Ten patients were on aspirin, 2 were on other antiplatelet drugs, and 1 was on both aspirin and clopidogrel. All aspirin and antiplatelet drugs were withheld during hospitalization. On discharge, aspirin and antiplatelet drugs were resumed in 7 patients taking them for secondary prevention of cerebro- or cardiovascular diseases but were withheld in 6 patients taking them for primary prevention of cerebro- or cardiovascular diseases.

One patient on warfarin for atrial fibrillation and aspirin for coronary artery disease had both medications withheld during hospitalization and restarted on discharge after hemostatic clipping.

#### Colonoscopy and DEP findings

Flat spots were found 75% in the TIC base and 25% at the neck (Figs. 2 and 3). In addition, 45.8% were in the left-sided colon and 54.2% in the right-sided colon. Twenty patients (83.3%) received colonoscopic hemostasis, all with hemostatic clipping. Of 19 patients who had DEP monitoring, 17 patients (89.5%) were positive for arterial blood flow underneath and later had hemostatic clipping (Figs. 1 and 2). One patient with negative arterial blood flow also had hemostatic clipping because of multiple TIC bleeds and prior partial colectomy. Of 5 patients without DEP monitoring, 2 patients had hemostatic clipping. One patient had fresh blood near the flat spot and fecalith within diverticulum. DEP could not be performed. In another patient, DEP was unavailable during urgent colonoscopy.

#### Rebleeding outcomes

Figure 5 shows a flow diagram of rebleeding outcomes. The cumulative rate (eg, early and late) of same TIC rebleeding in spot patients was 41.7% (10/24). Only 3 patients rebled within 30 days. One medical patient who did not have DEP monitoring or colonoscopic hemostasis rebled 2 days after the index bleed. He underwent urgent colonoscopy and computed tomography angiography (CTA), which did not reveal active bleeding or SRH. Two other patients had positive arterial blood flow and hemostatic clipping during the index bleed. One patient rebled 4 days later and underwent tagged RBC scan, CTA, and colonoscopy. No SRH was found. This patient rebled again 3 days afterward and had embolization due to positive CTA. Another patient rebled 8 days after index bleed and underwent embolization.

**Figure 5:**
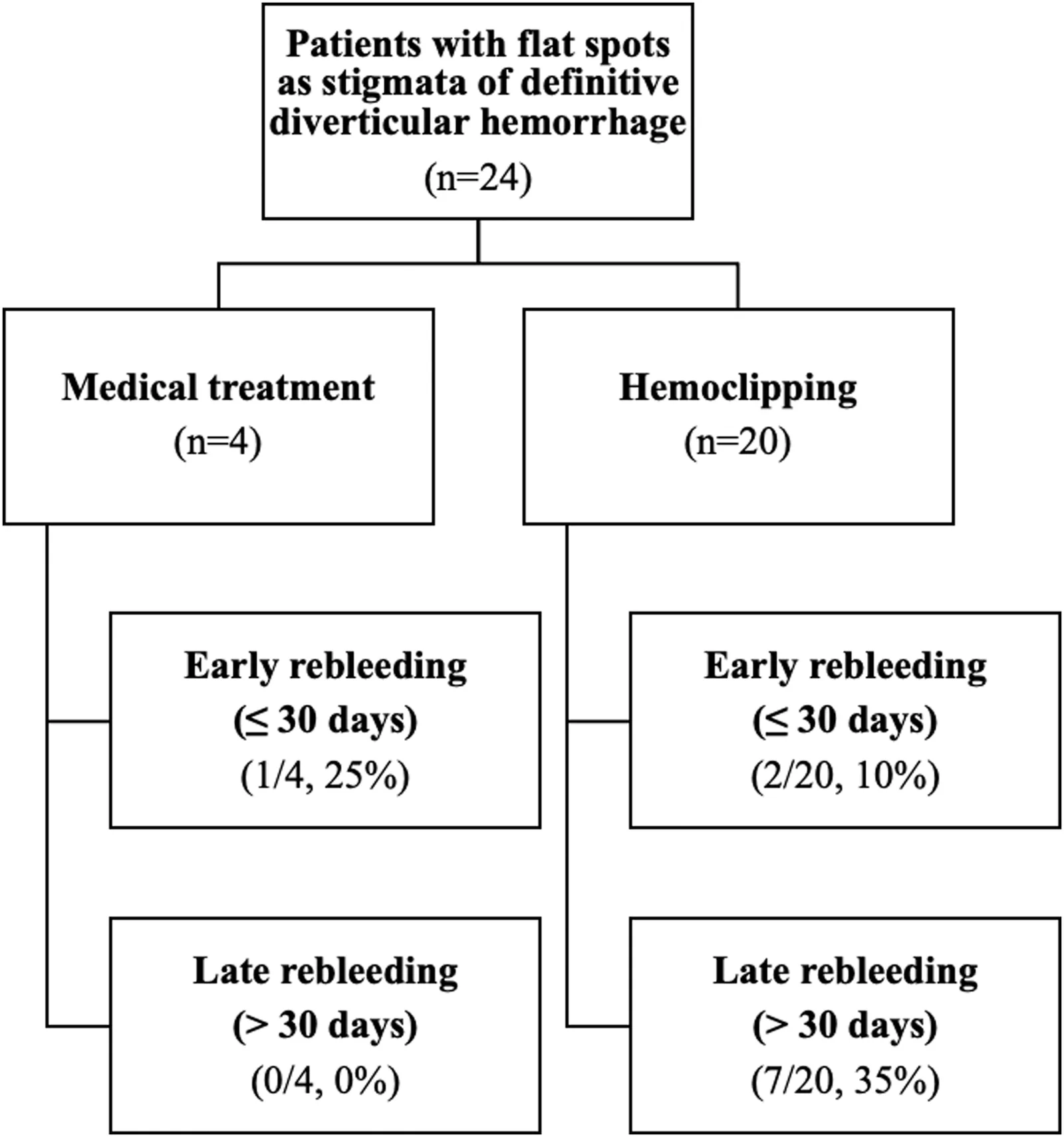
Flow diagram of rebleeding outcomes in patients with flat spots as stigmata of diverticular hemorrhage.

Of 7 patients with late rebleeding, 6 patients had DEP—5 positive and 1 negative arterial blood flow. One patient did not have DEP. However, all 7 patients received hemostatic clipping, and none had early rebleeding. During long-term follow-up, when all hemostatic clips had fallen off the TICs as documented on repeat urgent colonoscopy, the first 3 patients rebled once, were diagnosed with presumptive TICH, and treated medically. The fourth patient rebled once from definitive TICH and had repeat colonoscopic hemostasis. The fifth patient rebled from definitive TICH and had elective right hemicolectomy due to a history of multiple TICH. However, this patient later rebled from presumptive TICH and was treated medically. The sixth patient rebled from definitive TICH and underwent colonoscopy hemostasis and rebled again from presumptive TICH. The seventh patient rebled 3 times from definitive TICH, receiving colonoscopic hemostasis during the first episode and embolization during the third. Then, this patient had the fourth rebleeding episode diagnosed as presumptive TICH.

#### Other outcomes

There were no adverse events of urgent colonoscopy or hemostasis. During long-term follow-up with a median of 62 months, 2 patients (8.3%) developed diverticulitis: 1 from a jejunal TIC and another from a sigmoid TIC. The second patient had diverticulitis in the same colon segment as the TIC SRH on index colonoscopy, but this was 2 years after the index TICH and after the hemostatic clips fell off the TIC. One patient (4.2%) underwent laparoscopic right hemicolectomy due to multiple TIC rebleeds. All-cause mortality was 16.7%, but none was due to TICH.

#### Comparison of rebleeding outcomes in TICH from flat spots after medical versus endoscopic treatment

The cumulative rate of same TIC rebleeds was 20% higher in the endoscopic treatment group, but it was not significant (45% vs 25%, *P* = .615). The early same TIC rebleeding rate was 15% higher in the medical treatment group (25% vs 10%, *P* =.437). In contrast, the endoscopic treatment group had arithmetically higher rates of late same TIC rebleeds (35% vs 0%, *P* = .283; Table 1).

**Table 1:** Comparison of outcomes in diverticular hemorrhage from flat spots after medical versus endoscopic treatment

Doppler endoscopic probe use and outcomes	Medical treatment (n = 4)	Endoscopic treatment (n = 20)	*P* value
Doppler endoscopic probe, n (%)	1 (25)	18 (90)	**.018**
Positive Doppler result, n (% of patients with Doppler endoscopic probe monitoring)	0	17 (94.4)	.105
Total same TIC rebleeding, n (%)	1 (25)	9 (45)	.615
Early same TIC rebleeding (≤30 days), n (%)	1 (25)	2 (10)	.437
Late same TIC rebleeding (>30 days), n (%)	0	7 (35)	.283
Reintervention, n (%)	1 (25)	9 (45)	.615
Readmission, n (%)	1 (25)	7 (35)	1.000
Diverticulitis, n (%)	0	2 (10)	1.000
Surgery, n (%)	0	1 (5)	1.000
All-cause mortality, n (%)	0	4 (20)	1.000
Lost to follow-up, n (%)	2 (50)	4 (20)	.251

Boldface indicates significance (*P* < .05).

*TIC*, Diverticulosis.

Patients treated endoscopically had an arithmetically higher rate of antithrombotic use after the index TIC bleed than those treated medically (40% vs 25%, *P* = 1.000). For the endoscopic treatment group, 6 patients were on antiplatelet drugs, 1 patient on an anticoagulant, and 1 patient on both an antiplatelet drug and an anticoagulant. For the medical group, 1 patient was on an antiplatelet drug. No one was on an anticoagulant.

#### Comparison of rebleeding outcomes in TICH from flat spots at the base versus neck after endoscopic treatment

For 20 flat spot patients treated with hemostatic clips, 15 (75%) were at the base and 5 (25%) at the neck. Patients with flat spots at the base had similar rates of cumulative rebleeding as those at the neck (46.7% vs 40%, *P* = 1.000).

#### Comparison of DEP positivity and rebleeding rates in TICH from flat spots versus NBVVs

Forty-five patients with definitive TICH from NBVVs (Fig. 4) were compared to 24 TIC flat spot patients (Table 2). DEP use was significantly lower in the NBVV group (44.4% vs 79.2%, *P* = .006). However, for those with DEP monitoring, the rate of positive arterial blood flow was the same for the 2 groups (89.5% vs 90%), and all these were treated with colonoscopic hemostasis. Colonoscopic hemostasis was performed more in the NBVV group (97.8% vs 83.3%, *P* = .046).

**Table 2:** Comparison of clinical characteristics and outcomes of patients with diverticular hemorrhage from flat spots versus NBVVs

	TIC with flat spots (n = 24)	TIC with NBVVs (n = 45)	*P* value
Male gender, n (%)	18 (75)	32 (71.1)	.731
Age, years, mean (SD)	73 (10)	70 (13)	.292
Race, n (%)			**.039**
African American	8 (33.3)	15 (33.3)	
Asian	4 (16.7)	8 (17.8)	
Caucasian	8 (33.3)	22 (48.9)	
Hispanic	4 (16.7)	0	
Syncope, n (%)	2 (8.3)	3 (6.7)	1.000
Hypotension, n (%)	5 (20.8)	15 (33.3)	.276
Shock, n (%)	1 (4.2)	1 (2.2)	1.000
Onset of index bleed, n (%)			.657
Inpatient	1 (4.2)	5 (11.1)	
Outpatient	23 (95.8)	40 (88.9)	
Bleeding medications, n (%)			
Aspirin	12 (50)	23 (51.1)	.930
NSAIDs	4 (16.7)	9 (20)	1.000
COX-II inhibitor	0	2 (4.4)	.540
Anticoagulant	2 (8.3)	7 (15.6)	.480
Antiplatelet	3 (12.5)	6 (13.3)	1.000
Prior TICH, n (%)	13 (54.2)	9 (20)	**.004**
Glasgow-Blatchford Score, mean (SD)	8 (8)	8.5 (3.9)	.518
CURE Prognosis Score, median (IQR)	3 (1)	3 (2)	.492
Charlson Comorbidity Index, median (IQR)	4 (5)	3 (4)	.183
Lowest hemoglobin, g/dL, median (IQR)	8.3 (3)	8.5 (2.8)	.870
PRBC transfusion, n (%)	16 (66.7)	31 (68.9)	.850
No. of PRBC transfusion, median (IQR)	1 (3)	2 (4)	.114
Stigmata of hemorrhage location, n (%)			**.002**
Neck	6 (25)	29 (64.4)	
Base	18 (75)	16 (35.6)	
DEP, n (%)	19 (79.2)	20 (44.4)	**.006**
Positive Doppler result, n (% of patients with DEP)	17 (89.5)	18 (90)	1.000
Endoscopic therapy, n (%)	20 (83.3)	44 (97.8)	**.046**
Total same TIC rebleeding, n (%)	10 (41.7)	11 (24.4)	.139
Early same TIC rebleeding (≤30 days), n (%)	3 (12.5)	4 (8.9)	.687
Late same TIC rebleeding (>30 days), n (%)	7 (29.2)	7 (15.6)	.181
Reintervention, n (%)	10 (41.7)	14 (31.1)	.381
Readmission, n (%)	8 (33.3)	14 (31.1)	.850
Diverticulitis, n (%)	2 (8.3)	3 (6.7)	1.000
Surgery, n (%)	1 (4.2)	1 (2.2)	1.000
All-cause mortality, n (%)	4 (16.7)	19 (42.2)	**.036**
Lost to follow-up, n (%)	6 (25)	10 (22.2)	.795

Boldface indicates significance (*P* < .05).

*COX-II*, Cyclooxygenase 2; *CURE*, Center for Ulcer Research and Education; *DEP*, Doppler endoscopic probe; *IQR*, interquartile range; *NBVVs*, nonbleeding visible vessels; *NSAIDs*, nonsteroidal anti-inflammatory drugs; *PRBC*, packed red blood cell; *SD*, standard deviation; *TIC*, diverticulum; *TICH*, diverticular hemorrhage.

For those with SRH at the neck, 5 patients had flat spots and 29 patients had NBVVs. All neck flat spot patients were treated with hemostatic clips. For those with NBVVs, 62.1% were treated with MPEC or both MPEC and hemostatic clips, 34.5% with hemostatic clips, and 3.4% with an over-the-scope clip (OTSC) after standard through-the-scope hemostatic clips failed. The types of colonoscopic hemostasis were significantly different between the 2 groups (*P* = .024). The early rebleeding rate was 0% in spots and 10.3% in NBVVs at the neck (*P* = 1.000). The late rebleeding rate was 40% in spots and 6.9% in NBVVs (*P* = .094).

For those with SRH at the TIC base, 15 patients had flat spots and 15 patients had NBVVs. All patients with flat spot at the base were treated with hemostatic clips. For those with NBVVs, 73.3% were treated with hemostatic clips and 26.7% with MPEC or combined MPEC/hemostatic clips. The types of colonoscopic hemostasis were significantly different between the 2 groups (*P* = .032). Early rebleeding rates were 13.3% in spot patients and 0% in NBVV patients at the base (*P* = .483). The late rebleeding rate was 33.3% in spots and 33.3% in NBVVs (*P* = 1.000).

## Discussion

The rate of positive arterial blood flow underneath TIC flat spots was high: 89.5% of patients with DEP monitoring, the same as patients with NBVVs (90%). Overall, 83.3% of spot patients received hemostatic clips. However, 41.7% rebled from the same TIC, including 3 early and 7 late rebleeds. Patients treated medically had arithmetically higher rate of early rebleeds (*P* = .437). Those treated endoscopically had an arithmetically higher rate of late rebleeds (*P* = .283). Compared with TICs with NBVVs, patients with flat spots had significantly higher rates of DEP monitoring (*P* = .006) and lower rates of colonoscopic hemostasis (*P* = .0046). This is because colonoscopic hemostasis was recommended for all TICs with NBVVs,^5^ and 40% (18/45) of the NBVV cases were treated before the availability of colon DEP monitoring.

Previously TIC flat spots were thought by us to be minor SRH, but only a few patients were reported.^11^ Our current study included 24 patients with flat spots—a much greater number than our previous study. Clinically important was the high rate of rebleeding with hemostatic clipping: 10% within 30 days and 35% after 30 days.

Colonoscopy is the most effective treatment for definitive TICH. A cohort study in the United States reported that a very low rate of TIC rebleeding was observed in other major TIC SRH treated endoscopically.^5^ In comparison, rebleeding occurred in more than 50% of similar TIC patients treated medically.^6^ Similar results were reported in a multicenter study in Japan and a retrospective cohort study in the United States.^8^^,^^9^ Only 1 study of a small number of patients with flat spots reported rebleeding rates, with higher rebleeding rates in those treated medically than those treated endoscopically (20% vs 0%).^10^

Our current results indicate that patients treated medically had a 15% higher rate of early rebleeding than those treated endoscopically (25% vs 10%, *P* = .437). In contrast, 35% of those treated endoscopically had late same TIC rebleeding. However, this is probably a type II error because of the small number in the medical treatment group (n = 4). Moreover, the number of flat spots in the base were 3 times higher than in the neck, which may result in higher TIC rebleeding rates because the arteries are larger.^6^^,^^12^

What are the most likely reasons for the high total rebleeding rate of TIC spots after hemostatic clipping? First, 25% had TIC spots at the neck and 75% had TIC spots at the base. Spot location in the base increases the risk of rebleeding.^6^^,^^12^ The arteries in the base are larger than branches at the neck, and obliteration of blood flow with standard clips is technically more difficult and not always successful.^2^^,^^6^^,^^12^ For spots at the TIC neck, we now recommend MPEC because coaptive coagulation there is safe and effective.^12^ However, MPEC is not recommended for SRH at the base due to risks of perforation and postcoagulation syndrome.^12^

Secondly, hemostatic clipping is not a uniformly effective treatment because clips fall off (as documented by repeat colonoscopy with rebleeding) and do not always obliterate TIC arteries (as documented by later DEP monitoring).^12^ For spots and other SRH in the TIC base, better colonoscopic treatments such as a large OTSC or rubber band ligation are recommended for definitive hemostasis.^2^^,^^12^ These more often obliterate arterial blood flow under the SRH and also can obliterate the diverticulum.^2^^,^^12^ DEP in TICH can localize and trace the underlying artery, risk stratify, and facilitate definitive colonoscopic hemostasis by documenting obliteration of arterial blood flow under SRH.^12^^,^^14^^,^^15^

Third, there were arithmetically higher rates of long-term antithrombotic use in the spot patients with endoscopic treatment, which could increase the risk for late rebleeding.

The rates of arterial blood flow detection underneath major TIC SRH were previously reported as 100% in active bleeding, 93% in NBVVs, and 80% in adherent clots.^10^ In PUB, flat spots are considered a minor SRH because the rate of positive arterial blood flow with DEP is only 40.5%, and the rebleeding rate on medical therapy was about 20%.^16^ In contrast, our current TIC spot results are that 89.5% had positive arterial blood flow. This was the same as with TIC NBVVs. This along with high rebleed rates indicate that flat spots should be classified as a major SRH for TICH and treated with colonoscopic hemostasis.

Unlike with PUB, medical therapy such as proton pump inhibitors is not available to heal TICs and prevent rebleeding. Patients with flat spots had somewhat higher rates of early and late rebleeding than those with NBVVs. Some of the clinically relevant differences can be explained by a significant higher number and the type of colonoscopic hemostasis performed in the NBVV group: hemostatic clipping (47.7%), MPEC (43.2%), combined MPEC and hemostatic clipping (6.8%), and a large OTSC after hemostatic clipping failed (2.3%). Results may also relate to the significantly higher prevalence of spots in the TIC base than NBVVs (75% vs 35.6%).

This study has several limitations. First, there was a moderate number of TIC spot patients, which made statistical significance difficult to achieve after dividing into subgroups. Type II errors are evident, but trends still appear to be clinically relevant. Second, we could not perform multivariable analysis to account for confounders because of the small number of patients with spots in the medical group. Third, our hospitals are tertiary care centers, and our GI bleed team has training and expertise in the diagnosis and treatment of TICH that most other hospitals except in Japan commonly lack.^2^^,^^6^^,^^12^ Therefore, our study results and recommendations may not be generalizable to other hospitals, particularly in the United States, where urgent colonoscopy is neither recommended nor currently taught in most GI training programs.^17^ Fourth, rubber band ligation was not performed for TIC spots, and an OTSC was used in only 1 patient.^2^ Further study of these treatments may significantly lower TIC rebleeding rates particularly for SRH in the TIC base.^2^^,^^12^ MPEC treatment of TIC spots at the neck could also lower rebleeding rates, similar to NBVVs.^12^

The strengths of this study are that we only included patients with TICH from flat spots, except when these were compared with NBVV cases. There was a significantly larger number of patients than in any previous study. We also reported rates of positive arterial blood flow detection with DEP in patients and compared clinical outcomes based on treatments and flat spot location in the TIC and regions of the colon. We also included a comparison with NBVVs, a recognized major SRH of TICH. Similar results have not been previously reported.

In summary, flat spots found during TICH should be considered as a major SRH and treated with colonoscopic hemostasis. This is due to high rates of arterial blood flow detected underneath TIC spots, the potential of rebleeding on medical treatment alone, and the high rate of late rebleeding with standard through-the-scope hemostatic clipping. Including flat spots as a major SRH has the potential of increasing the diagnostic rate of definitive TICH during urgent colonoscopy by about 10%.^12^ Further studies are warranted to determine the best colonoscopic hemostasis methods for long-term definitive hemostasis of flat spots both at the TIC base and neck.

## Financial support

This study was supported by a Veterans Administration Clinical Merit Review Research Grant (grant number 5I01CX001403) and the 10.13039/100000062National Institutes of Health–National Institute of Diabetes and Digestive and Kidney Diseases, CURE Digestive Diseases Research Center Human Studies Core (grant number P30DK41301).

## Patient consent

All patients provided written informed consent and were enrolled in studies of severe hematochezia conducted in accordance with the Institutional Review Board approval and ethical guidelines.

## Disclosure

All authors disclosed no financial relationships.
